# Accelerated free-breathing volumetric liver proton density fat fraction (PDFF) and ${\mathbf{R}}_{2}^{\mathbf{*}}$ quantification in pediatric patients using stack-of-radial MRI with multidimensional regularized reconstruction: a retrospective study

**DOI:** 10.1007/s10334-026-01333-y

**Published:** 2026-02-19

**Authors:** Xiaodong Zhong, Marcel D. Nickel, Brian M. Dale, Cara E. Morin, Zachary Abramson, Yuxi Pang, Fei Han, Vibhas Deshpande, Stephan A. R. Kannengiesser, Aaryani Tipirneni-Sajja

**Affiliations:** 1 Department of Radiological Sciences, David Geffen School of Medicine, University of California Los Angeles, 300 Medical Plaza Drive, Suite B119B, Los Angeles, CA 90095-7350 USA; 2 Physics and Biology in Medicine Interdepartmental Program, David Geffen School of Medicine, University of California Los Angeles, Los Angeles, CA USA; 3 Department of Bioengineering, Samueli School of Engineering, University of California Los Angeles, Los Angeles, CA USA; 4 MR Application Predevelopment, Siemens Healthineers AG, Forchheim, Germany; 5 MR R&D Collaborations, Siemens Medical Solutions USA, Inc, Cary, NC USA; 6 Department of Radiology, Cincinnati Children’s Hospital Medical Center, Cincinnati, OH USA; 7 Department of Diagnostic Imaging, St. Jude Children’s Research Hospital, Memphis, TN USA; 8 MR R&D Collaborations, Siemens Medical Solutions USA, Inc, Los Angeles, CA USA; 9 MR R&D Collaborations, Siemens Medical Solutions USA, Inc, Austin, TX USA; 10 Department of Biomedical Engineering, Department of Biomedical Sciences, University of Houston, Houston, TX USA

**Keywords:** Pediatric MRI, PDFF, ${\mathrm{R}}_{2}^{*}$, Respiratory motion compensation, Compressed sensing, Multidimensional regularization, Radial MRI

## Abstract

**Objective:**

To evaluate an accelerated free-breathing three-dimensional stack-of-radial MRI technique of liver proton density fat fraction (PDFF) and ${\mathrm{R}}_{2}^{*}$ quantification on retrospectively undersampled pediatric patient data.

**Methods:**

A newly developed compressed sensing reconstruction with multidimensional regularization was evaluated on undersampled data in 21 clinical pediatric subjects at 1.5 T with respect to the reference-standard self-gating reconstruction on oversampled data (700 radial views per slice). PDFF and ${\mathrm{R}}_{2}^{*}$ maps were calculated using the proposed method with fully sampled and undersampled radial views (352 and 176) and compared to reference-standard results using Bland-Altman analysis (reported as [mean difference; lower, upper limits of agreement]). Practical equivalence was evaluated using Bayesian posterior analysis with region of practical equivalence (ROPE) at ± 3% for PDFF and ± 10 s^−1^ for ${\mathrm{R}}_{2}^{*}$.

**Results:**

Echo images, PDFF and ${\mathrm{R}}_{2}^{*}$ maps reconstructed using the proposed method had reduced image artifacts. Bland-Altman plots showed that the proposed method using 176 views had lower biases, [-0.7;-2.4,1.0]% for PDFF and [-5.3;-25.5,14.9] s^−1^ for ${\mathrm{R}}_{2}^{*}$, compared to the reference method using 352 views: [0.8; -0.6, 2.1]% for PDFF and [-9.2;-51.9,33.4] s^−1^ for ${\mathrm{R}}_{2}^{*}$. Bayesian posterior analysis revealed that our proposed method using 176 views was practically equivalent to the reference method using 700 views with 100% within the PDFF ROPE and with 98.25% within the ${\mathrm{R}}_{2}^{*}$ ROPE. An acceleration factor of 4 and an approximate acquisition time saving of 67% shorter could be achieved.

**Conclusion:**

The proposed method may allow accelerated free-breathing liver PDFF and ${\mathrm{R}}_{2}^{*}$ mapping in pediatric subjects in approximately 1 min.

## Introduction

Hepatic steatosis is a condition with excessive liver fat deposition and a central characteristic of liver diseases such as metabolic dysfunction-associated steatotic liver disease (MASLD), the most common chronic liver disease in children and adults [1–4]. In addition, liver iron deposition is a critical condition in children and adults with hemochromatosis, and in those undergoing repeated blood transfusions [5, 6]. Non-invasive quantitative magnetic resonance imaging (MRI) plays an important role in measuring liver fat and iron levels for diagnosis, staging, and monitoring during treatment [7–10].

Although breath-hold three-dimensional (3D) multi-echo Cartesian gradient-echo (GRE) imaging is a well-validated MRI method for liver fat (given by proton density fat fraction, PDFF) and iron quantification (expressed by the effective transverse relaxation rate, ${\mathrm{R}}_{2}^{*}$) in adults [11–20], it is challenging to implement in children who have difficulty with breath-holding. Alternatively, 3D stack-of-radial imaging can provide accurate PDFF and ${\mathrm{R}}_{2}^{*}$ quantification in both adult and pediatric patients using free-breathing acquisitions [21–26], if combined with self-gating techniques, which accepts only radial views within one respiratory motion state and thus corrects measured ${\mathrm{R}}_{2}^{*}$ biases [23–26]. The self-gating stack-of-radial approach has also been validated for liver iron quantification against a 2D biopsy-calibrated ${\mathrm{R}}_{2}^{*}$ relaxometry method in pediatric patients [26]. However, after self-gating, only a subset of radial views is used for reconstruction, causing possible residual undersampling artifacts and increased noise. This often necessitates prolonged acquisition (e.g. 1.75–2 times longer) of additional radial views (i.e. oversampled) [23–26]. Based on compressed sensing (CS) techniques [27–30], a recent study proposed to address this limitation using self-gating to resolve all acquired radial views into multiple respiratory motion states in conjunction with CS reconstruction with motion-resolved multidimensional regularization [31]. This new technique was validated in phantoms and adult subjects [31], where it saved 61–65% of equivalent acquisition time compared to the protocol with fully sampled radial views and self-gating [23–26]. However, the performance of this technique still needs to be investigated in pediatric subjects to demonstrate feasibility for clinical adoption.

In this study, our purpose was to utilize retrospective virtual data acquisition acceleration to evaluate our proposed free-breathing stack-of-radial liver PDFF and ${\mathrm{R}}_{2}^{*}$ quantification technique with motion-resolved multidimensional regularized CS reconstruction [31] in pediatric patients, with reference to oversampled data from a previous study [26].

## Material and methods

### CS reconstruction with multidimensional regularization

In this study, we reconstruct undersampled stack-of-radial data by a CS approach to minimize a cost function $\frac{1}{2}{\Vert Ax-y\Vert }_{2}^{2}+{\Vert W(\alpha ,\beta )x\Vert }_{1}$, where A is a catch-all matrix collectively applying all relevant operations consisting of multiplication with coil sensitivity maps and non-uniform Fourier transformation; x is a 3D image matrix (2D spatial + 1D motion-state) to be reconstructed for each slice; y is the acquired k-space data assigned to k-space and motion-state dimensions; $W(\alpha ,\beta )$ is the redundant Haar wavelet transformation applied in in-plane spatial and motion-state dimensions whose low- and high-pass filters are separately scaled by individual regularization constants α and β; ${\Vert \cdot \Vert }_{2}$ is the Euclidean ${{\ell}}_{2}$-norm, and ${\Vert \cdot \Vert }_{1}$ is the ${{\ell}}_{1}$-norm. More details can be found in the reference [31].

### In vivo* data*

In a previous study [26], raw data were obtained with approval from the local institution review board and were retrospectively processed offline for new analysis in this study. This study was compliant with the Health Insurance Portability and Accountability Act. It was approved by the Institutional Review Board of St. Jude Children’s Research Hospital, which waived the requirement for informed consent.

The previous study [26] was performed at 1.5 T (MAGNETOM Avanto^fit^, Siemens Healthineers, Forchheim, Germany) for liver iron overload evaluation, from which 21 clinical pediatric subjects without global fat/water swapping were included for initial analyses (15 females, 14.7 ± 4.2 yrs, body mass index [BMI]: 24.8 ± 6.4 kg/m^2^). For additional analyses, 17 subjects were used due to incomplete availability of some original datasets (11 females, 15.2 ± 4.2 years, BMI: 24.7 ± 4.4 kg/m^2^). The number of subjects included in each analysis and figure is provided in the Methods and Results sections. The acquisition parameters are listed in Table 1.

**Table 1:** Imaging parameters and time saving of the protocols used in this study

Imaging parameters	Free-breathing stack-of-radial
Field strength (T)	1.5
TR (ms)	8.85 or 8.34
The 1st TE (ms)	1.19–1.23
ΔTE (ms)	1.24 or 1.19
Number of echoes	6
FOV (mm^2^)	320 × 320–380 × 380
Image matrix	224 × 224
In-plane pixel size (mm^2^)	1.4 × 1.4—1.7 × 1.7
Through-plane pixel size (mm)	5
Number of slices	30–48
Flip angle (degree)	5
Gradient readout mode	Bipolar
Imaging orientation	Axial
Slice resolution (%)	100 or 50
Slice oversampling (%)	18–50
Original: acquired radial views per slice (equivalent sampling factor)	700 (2.0)
Original: total acquisition time (seconds)	144–417
Proposed: virtually equivalent total acquisition time for 176 radial views per slice (sampling factor = 0.5) (seconds) (Equivalent percentage of time saving compared to the original acquisition time)	47 (67% shorter) - 137 (67% shorter)

### Raw data undersampling processing

All raw data in this study were acquired using a multi-echo GRE stack-of-radial research application sequence [21–26] with golden-angle ordering [29, 30] and 700 radial views per slice in k-space (equivalent to a sampling factor of 2, oversampled) [26]. Raw data were undersampled to 352 and 176 radial views (sampling factors 1.0 and 0.5, respectively) for further image reconstruction and processing in 21 subjects.

A custom script in Python (v.3.10.4, Python Software Foundation, Delaware, USA) using the twixtools package [32] was implemented to undersample a specific number of radial views in one raw data file acquired with oversampled, 700 radial views and save as a new raw data file for processing. Undersampling was achieved by keeping a specified beginning portion of acquired radial views in a raw data file.

The sampling factor of a raw data set was calculated as the ratio of radial views to the threshold, fully sampled radial views determined by the Nyquist k-space sampling criterion (i.e. $image matrix\times \frac{\pi }{2}$) [29], which corresponds to approximately 352 in this study.

### Image-reconstruction methods for comparison

The image reconstruction pipeline includes data-processing steps of k-space shift correction [21–26, 33], self-gating [23, 24], multidimensional regularization [31], and adaptive fitting for PDFF and ${\mathrm{R}}_{2}^{*}$ quantification [16]. This was implemented in C + + and integrated into the vendor-provided research application sequence and image reconstruction framework (Siemens Healthineers, Forchheim, Germany) for retrospective offline reconstruction using raw data files. The implementation was capable of utilizing graphics processing units (GPUs) (if available), allowing for more rapid image reconstruction compared to reconstruction based only on the central processing unit (CPU) [31]. Resultant echo images and PDFF and ${\mathrm{R}}_{2}^{*}$ maps were generated and stored in the format of Digital Imaging and Communications in Medicine (DICOM).

Two methods were applied to the raw data of 21 subjects with 352 and 176 radial views (sampling factors 1.0 and 0.5, respectively) to reconstruct images for comparison. First, as an established and current method, self-gating was utilized to accept only radial views acquired near end-expiration which accounted for 40% of all radial views, followed by nonuniform fast Fourier transform (termed as the “stable40” method). Specifically, as validated in the previous study [26], images and PDFF and ${\mathrm{R}}_{2}^{*}$ results of the stable40 method based on the originally acquired oversampled data with 700 radial views (i.e. a sampling factor of 2) were used as the reference standard [23–26]. In addition, results of the “proposed” method were obtained as described in Sect. “CS reconstruction with multidimensional regularization” on respiratory motion resolved data with 4 respiratory motion states [31]. The regularization constants were set empirically according to the previous study [31].

Two additional analyses were performed in 17 subjects. First, to further evaluate the effect of undersampling, PDFF and ${\mathrm{R}}_{2}^{*}$ mean values within the regions of interest (ROIs) were calculated for each subject at varying numbers of views (50–700 in steps of 50) and compared to the results at 700 views as a reference. In addition, PDFF and ${\mathrm{R}}_{2}^{*}$ mean values measured at three respiratory motion states corresponding to 38%, 63%, and 88% of the respiratory phase respectively (approximately representing early-, mid- and end-inspiration) were compared to the reference values at end-expiration.

### Statistical analysis

The same whole-liver ROIs on a mid-level slice for all subjects from the previous study were used, where blood vessels were excluded based on a ${\mathrm{R}}_{2}^{*}$ thresholding approach [26]. PDFF and ${\mathrm{R}}_{2}^{*}$ values within the whole-liver ROIs excluding blood vessels were measured as mean ± standard deviation (SD). Using reference results of the stable40 method on data with 700 radial views, Bland–Altman analysis was performed to determine mean differences (MD) and limits of agreement (LoA = MD ± 1.96 × SD) of different methods, reported as [MD; lower LoA, upper LoA] to evaluate the results at 352 and 176 radial views in 21 subjects.

In addition, in 21 subjects, a Bayesian model was computed with $Y\sim \mathcal{N}({\mu }_{i},{\sigma }_{i})$ where Y is PDFF or ${\mathrm{R}}_{2}^{*}$ and i indicates the reconstruction method. ${\sigma }_{i}$ accounted for heteroskedasticity in the data. A posterior analysis was performed to test practical equivalence of other methods using reference results of the stable40 method on data with 700 radial views. A region of practical equivalence (ROPE) was set at ± 3% for PDFF and ± 10 s^−1^ for ${\mathrm{R}}_{2}^{*}$. Based on prior experience, such variations are well within the normal variation of healthy individuals, where the variation is the least. The Bayesian analysis was performed using R version 4.4.1 (R Foundation for Statistical Computing, Vienna, Austria) using the brms and bayestestR packages [34, 35].

To evaluate the undersampling effect on PDFF and ${\mathrm{R}}_{2}^{*}$ at 50–700 views in steps of 50, in 17 subjects, three types of plots were generated: (1) mean vs. views with shaded areas representing ± 0.5 × SD across subjects to illustrate inter-subject variability, (2) SD vs. views to quantify inter-subject variability directly, and (3) absolute percentage deviation from the 700-view reference mean to assess diminishing returns, with the elbow point defined as the smallest number of views at which the deviation falls below a predefined threshold. For PDFF, the threshold was set at 15%, corresponding approximately to an absolute PDFF difference of 3% based on a representative grade 3 pediatric steatosis PDFF of 23.3% [36]. For ${\mathrm{R}}_{2}^{*}$, the threshold was set at 3%, corresponding approximately to an absolute ${\mathrm{R}}_{2}^{*}$ difference of 12–15 s^−1^, considering a ${\mathrm{R}}_{2}^{*}$ value of about 400–500 s^−1^ as moderate to severe pediatric liver iron overload [37].

Boxplot analysis of respiratory motion effects was performed in 17 subjects. Subject-wise differences were computed for each non-reference respiratory state using end-expiration results as a reference. These paired differences were summarized using boxplots to visualize the distribution of motion-induced deviations across subjects.

### Equivalent acquisition times

To account for the time of other sequence components, the equivalent acquisition times corresponding to the protocols with different sampling factors were recorded using the vendor-provided sequence simulation tool (Siemens Healthineers, Forchheim, Germany).

## Results

### Representative undersampled data and self-gating of two methods

**Fig. 1:**
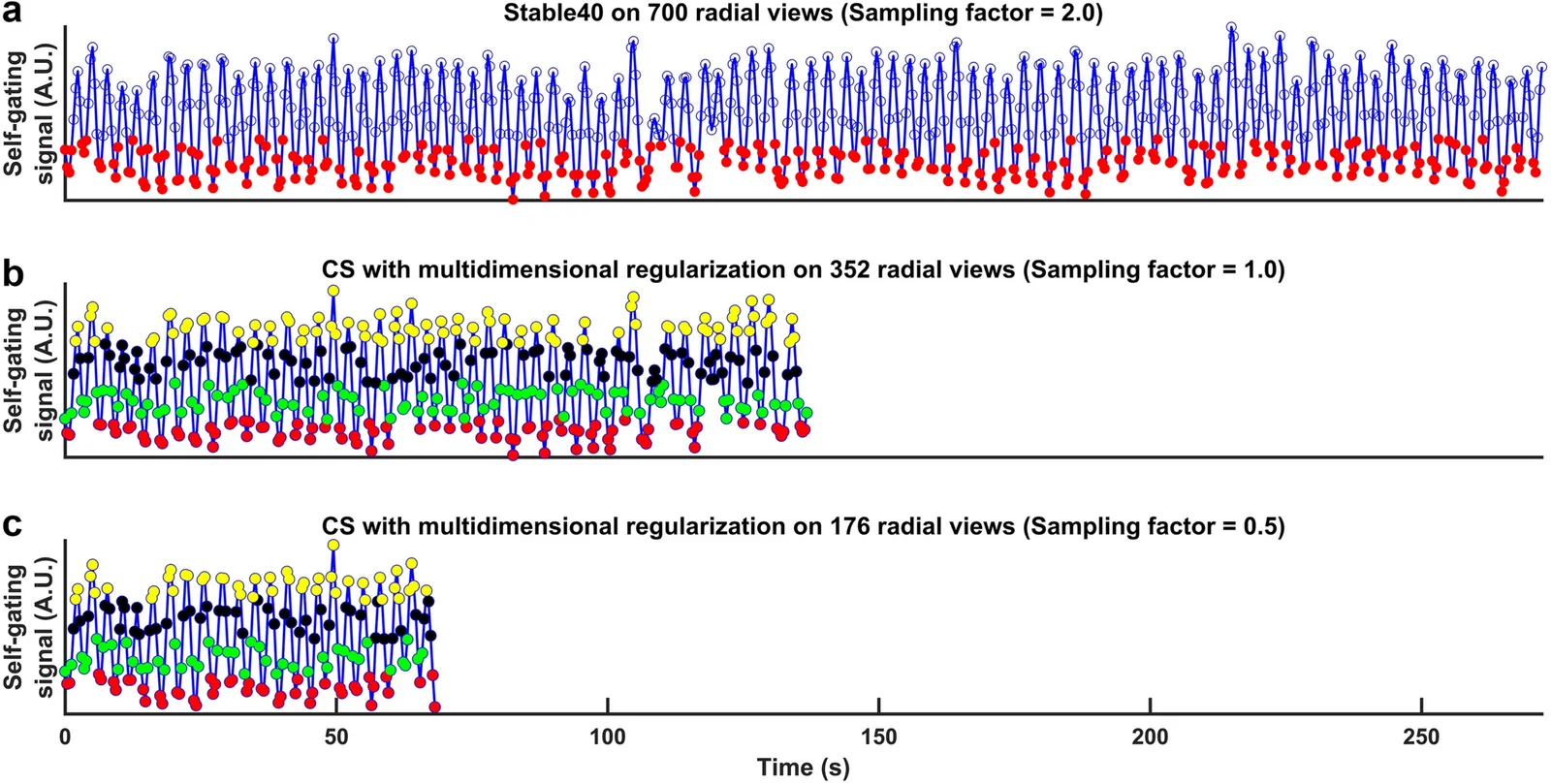
Self-gating signal curves extracted from free-breathing radial raw data of one 10-year-old subject with different radial view sampling factors. Data with the sampling factor of 2.0 (700 radial views) was originally acquired (**a**), where red dots represent the radial views selected by the stable40 method for reconstruction. An example of 4 respiratory motion states is shown by markers with different colors in undersampled data generated retrospectively with sampling factors of 1.0 (**b**) and 0.5 (**c**), used for CS reconstruction with multidimensional regularization

Representative self-gating signal curves from the raw data of one subject are shown in Fig. 1 to demonstrate the respiratory gating and binning strategies in this study. In Fig. 1a, a total of 40% of data close to end-expiration are identified and accepted by the stable40 method on the reference data with 700 radial views (red dots in Fig. 1a). Figure 1b and c demonstrates that data generated retrospectively with 352 (Fig. 1b) and 176 radial views (Fig. 1c) are resolved into 4 respiratory motion states for the proposed method (CS reconstruction with multidimensional regularization).

### *Representative echo images, PDFF maps and *${\mathrm{R}}_{2}^{*}$* maps*

**Fig. 2:**
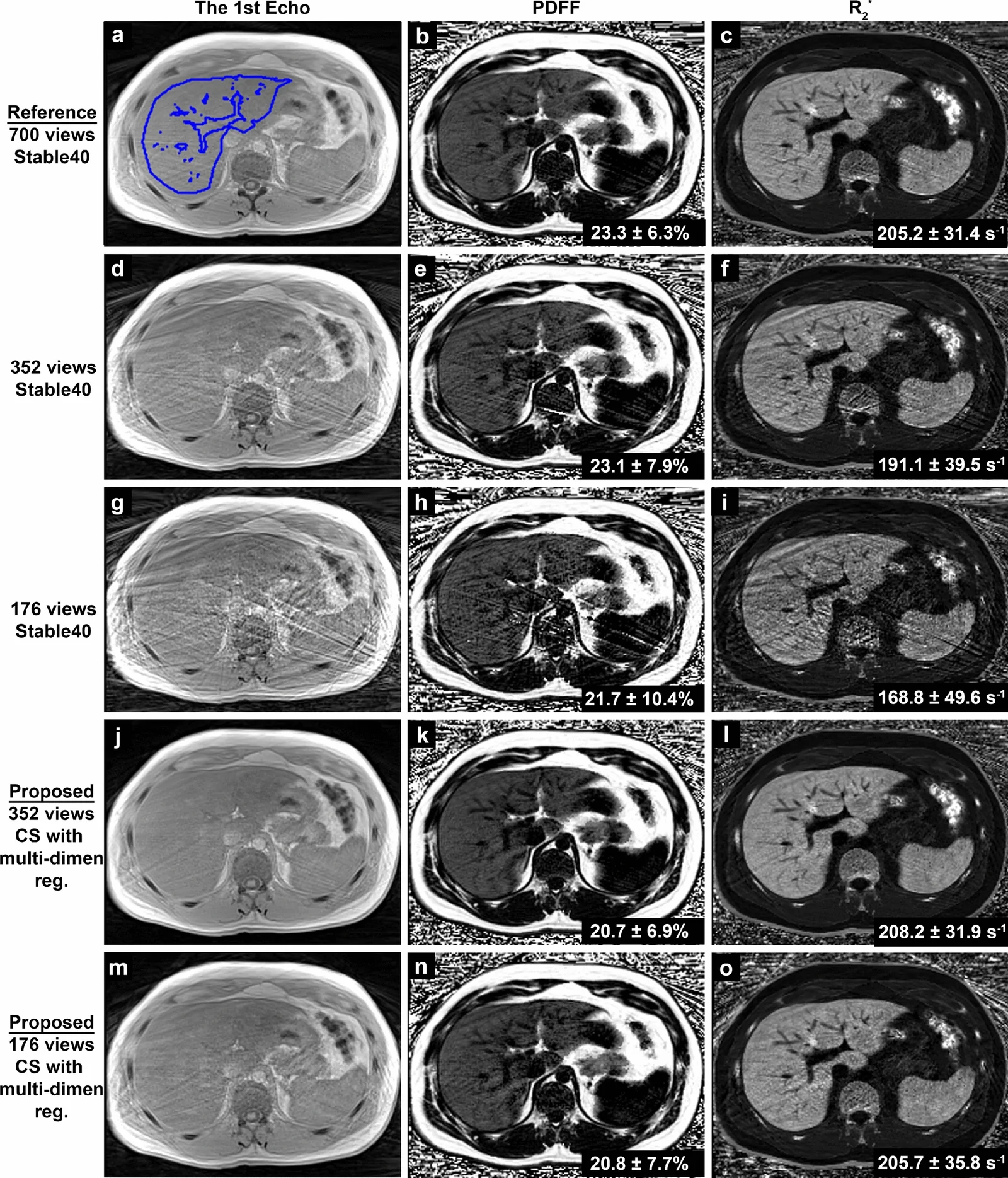
Example echo images, PDFF and ${\mathrm{R}}_{2}^{*}$ maps of a 23-year-old subject with high liver fat and iron deposition reconstructed with the stable40 (**a-i**) and proposed methods (**j-o**) using data with 700, 352 and 176 views. Reference results are from the stable40 method on data with 700 views (**a-c**). All methods show results at the first motion state (end-expiration). Images and maps are cropped for better visualization. PDFF and ${\mathrm{R}}_{2}^{*}$ values within the whole-liver contour excluding blood vessels (indicated by the blue outline in a) are reported as mean ± SD

Example echo images, PDFF and ${\mathrm{R}}_{2}^{*}$ maps of one subject with moderate liver fat and iron deposition are shown in Fig. 2. The results reconstructed with the stable40 and proposed methods using data with 352 and 176 views (sampling factor = 1.0 and 0.5, respectively) are shown in Fig. 2d-o and compared to reference results from the stable40 method on data with 700 radial views (Fig. 2a-c). Streaking artifacts are evident on echo images, PDFF and ${\mathrm{R}}_{2}^{*}$ maps using the stable40 method on data with 352 or 176 radial views (Fig. 2d-i). Reduced streaking artifacts on echo images, PDFF and ${\mathrm{R}}_{2}^{*}$ maps using the proposed method are observed (Fig. 2j-o), and PDFF (Fig. 2k, n) and ${\mathrm{R}}_{2}^{*}$ measurements (Fig. 2l, o) match the reference results (Fig. 2b, c).

**Fig. 3:**
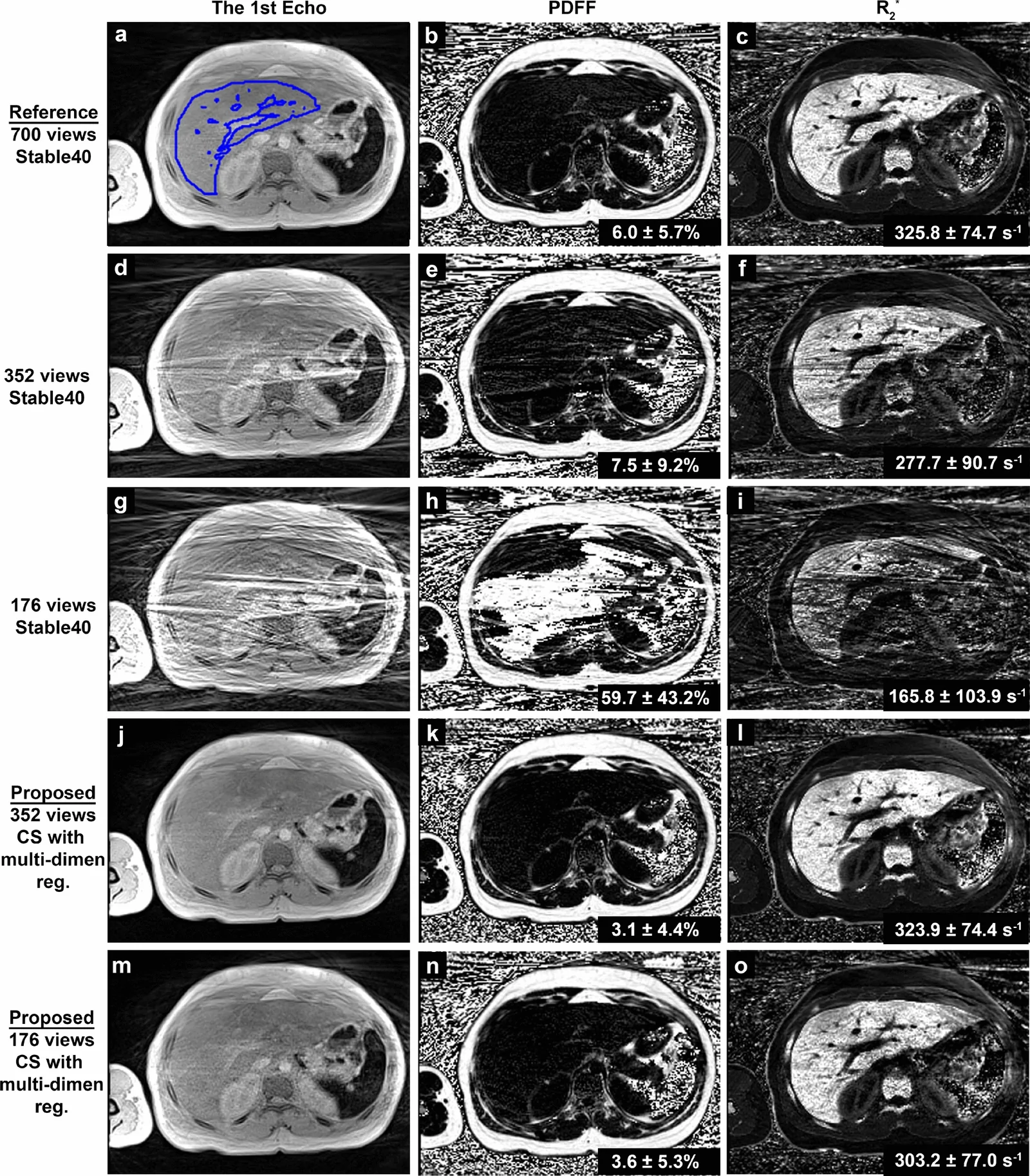
Example echo images, PDFF and ${\mathrm{R}}_{2}^{*}$ maps of a 13-year-old subject with relatively high liver iron deposition reconstructed with the stable40 (**a-i**) and proposed methods (**j-o**) using data with 700, 352 and 176 views. Undersampling streaking artifacts are pronounced on echo images of the stable40 method using data with 176 views (**g**), accompanied by local fat/water swapping problems on the PDFF map (**h**) and aberrations on the ${\mathrm{R}}_{2}^{*}$ map (**i**), leading to erroneous PDFF and ${\mathrm{R}}_{2}^{*}$ values. All methods show results at the first motion state (end-expiration). Images and maps are cropped for better visualization. PDFF and ${\mathrm{R}}_{2}^{*}$ values within the whole-liver contour excluding blood vessels (indicated by the blue outline in a) are reported as mean ± SD

Results of another subject with relatively high liver iron deposition are shown in Fig. 3. Pronounced streaking artifacts are observed on echo images of the stable40 method using data with 176 views (Fig. 3g), which causes local fat/water swapping problems on the PDFF map (Fig. 3h) and aberrations on the ${\mathrm{R}}_{2}^{*}$ map (Fig. 3i), leading to erroneous PDFF and ${\mathrm{R}}_{2}^{*}$ values. Reduced artifacts on images and more accurate maps using the proposed method are observed (Fig. 3j-o).

### Statistical analysis

The ranges of PDFF and ${\mathrm{R}}_{2}^{*}$ in this pediatric cohort were 2.0%–23.3% and 46.8 s^−1^–497.9 s^−1^ respectively, according to the stable40 reference results on data with 700 views.

**Fig. 4:**
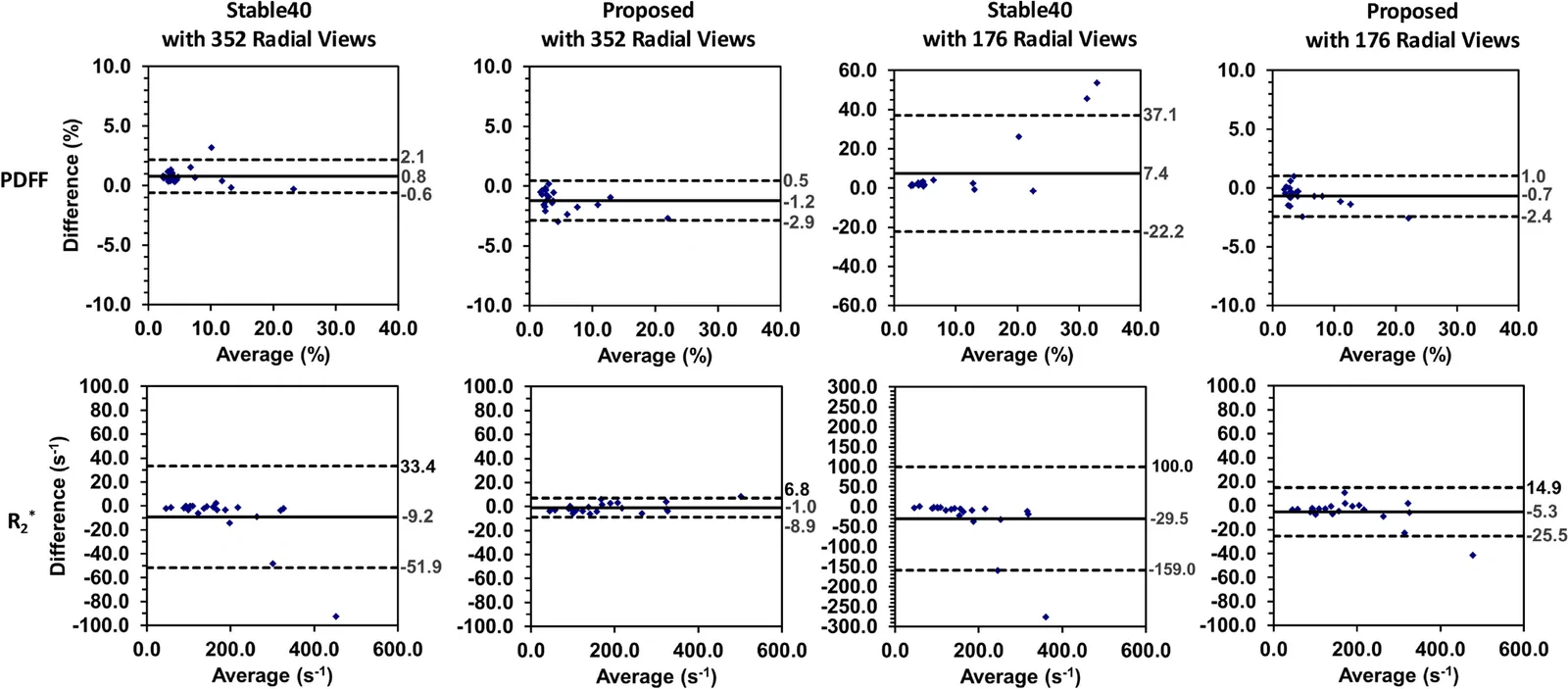
Bland–Altman plots comparing PDFF (**a-d**) and ${\mathrm{R}}_{2}^{*}$ (**e–h**) results from the stable40 method (**a, c, e, g**) and the proposed method (**b, d, f, h**) vs. reference results from the stable40 method on data with 700 views in 21 subjects

Comparing reference PDFF and ${\mathrm{R}}_{2}^{*}$ results from the stable40 method on data with 700 views, Bland–Altman plots of the stable40 and proposed methods on data with 352 and 176 views in 21 subjects are shown in Fig. 4. MD and LoA of the proposed method on data with 176 views were [−0.7; −2.4, 1.0]% for PDFF, similar to those of the stable40 method on data with 352 radial views: [0.8; −0.6, 2.1]%. For ${\mathrm{R}}_{2}^{*}$, MD and LoA of the proposed method on data with 176 views were [−5.3; −25.5, 14.9] s^−1^, lower than MD and LoA of the stable40 method on data with 352 radial views: [−9.2; −51.9, 33.4] s^−1^.

In the equivalence test with Bayesian posterior analysis in 21 subjects, it was found that stable40 on data with 176 views was not practically equivalent to the reference stable40 method on data with 700 views, with only 13.45% of the posterior distribution of expected mean PDFF being within ROPE, and all other method were practically equivalent with 100% within ROPE. As for ${\mathrm{R}}_{2}^{*}$, the proposed method on data with both 176 and 352 views were practically equivalent to the reference method, with 98.25% and 100%, respectively, of their posterior distribution within ROPE, while neither stable40 on data with 176 views nor stable40 on data with 352 views were practically equivalent, with 25.12% and 66.95%, respectively, within ROPE.

**Fig. 5:**
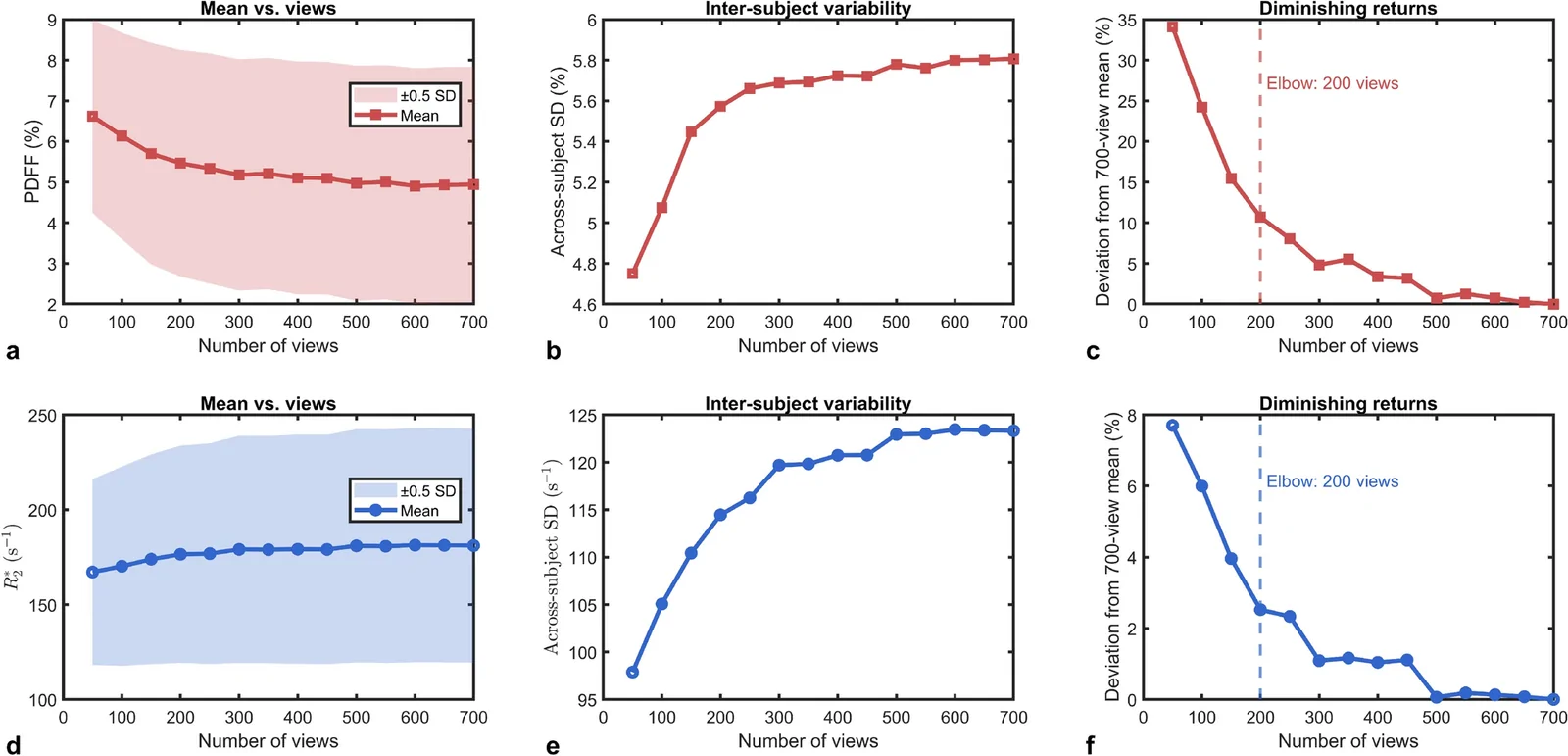
Plots illustrating the effect of undersampling on PDFF (the top row) and ${\mathrm{R}}_{2}^{*}$ (the bottom row) across 50–700 views in steps of 50 in 17 subjects. (**a, d**): Mean vs. views with shaded areas representing ± 0.5 × SD across subjects to illustrate inter-subject variability. (**b, e**): SD vs. views to quantify inter-subject variability directly. (**c, f**): Absolute percentage deviation from the 700-view reference mean to assess diminishing returns, with the elbow point defined as the smallest number of views at which the deviation falls below a predefined threshold

The plots illustrating the effect of undersampling on PDFF and ${\mathrm{R}}_{2}^{*}$ across 50–700 views in steps of 50 in 17 subjects are shown in Fig. 5. PDFF mean decreased from ~ 7% to 5% (Fig. 5a), while R₂* mean increased from ~ 170 to 180 s^−1^ (Fig. 5d). Inter-subject SD increased modestly for PDFF (~ 4.7% to 5.8%, Fig. 5b) and more substantially for ${\mathrm{R}}_{2}^{*}$ (~ 97 to 123 s^−1^, Fig. 5e). Elbow points were observed at ~ 200 views for both PDFF (Fig. 5c) and ${\mathrm{R}}_{2}^{*}$ (Fig. 5f), indicating the approximate minimum number of views needed to reach near-reference accuracy.

**Fig. 6:**
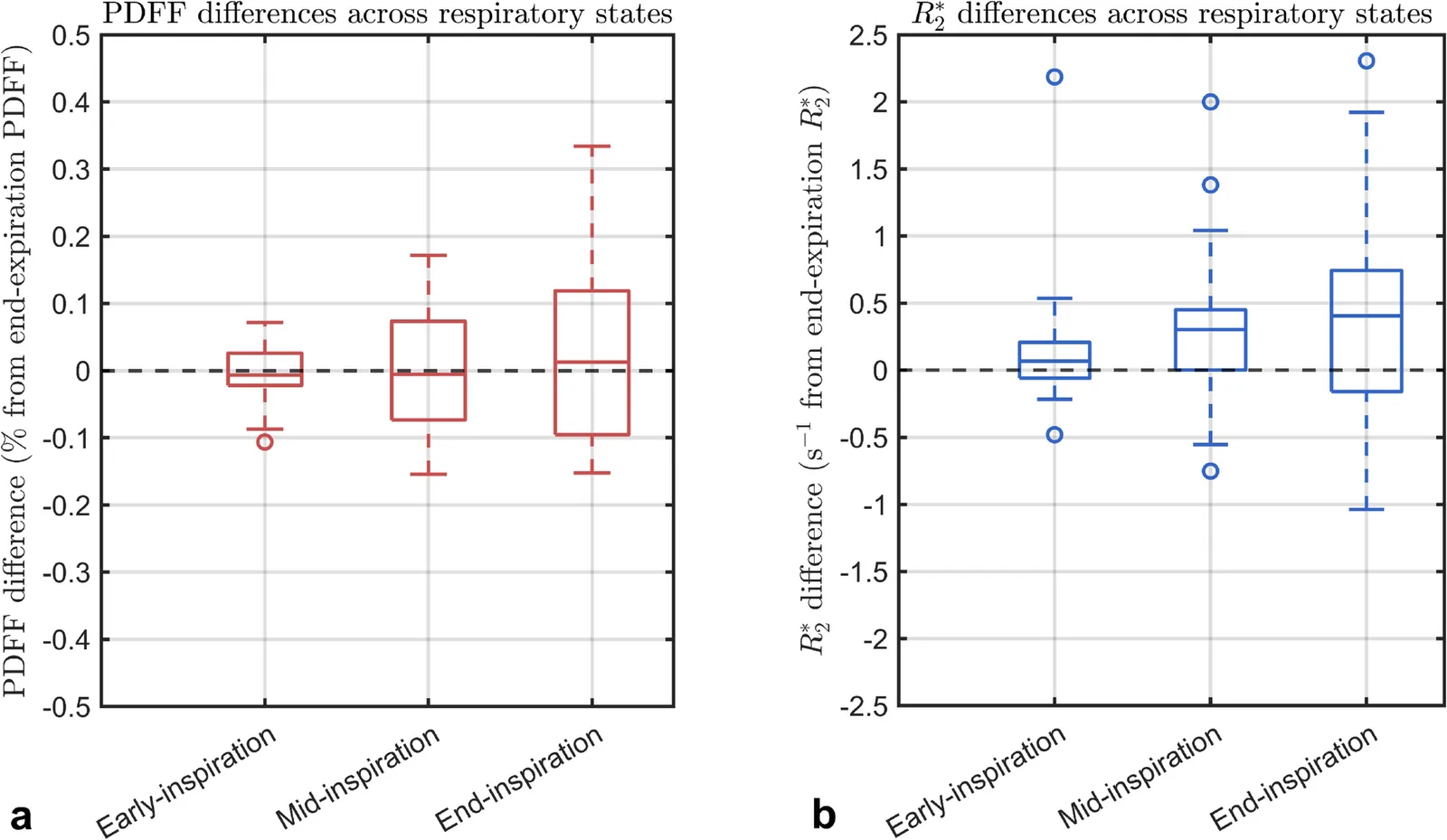
Boxplots of PDFF and ${\mathrm{R}}_{2}^{*}$ differences relative to end-expiration demonstrated motion-dependent deviations across 17 subjects. In each boxplot, the central line represents the median difference, the box spans the interquartile range (IQR; 25th-75th percentiles), and the whiskers extend to the most extreme values within 1.5 × IQR. Values beyond this range are shown as individual open circles and represent statistical outliers. A horizontal dashed line at zero indicates no difference relative to end-expiration

Boxplots of PDFF and ${\mathrm{R}}_{2}^{*}$ differences relative to end-expiration demonstrating respiratory motion-state dependent deviations across 17 subjects are shown in Fig. 6. For PDFF, the distributions showed modest shifts across respiratory states, with median differences remaining close to zero and relatively narrow interquartile ranges, indicating negligible sensitivity to respiratory motion. For ${\mathrm{R}}_{2}^{*}$, larger spreads were observed, suggesting slightly greater variability in ${\mathrm{R}}_{2}^{*}$ measurements with respiratory state. In both PDFF and ${\mathrm{R}}_{2}^{*}$, most differences fell within the whisker range, while a small number of open circles indicated outlier subjects exhibiting larger deviations from the reference state. The zero-reference line allowed direct visual assessment of both the direction and magnitude of motion-induced bias.

### Equivalent acquisition time saving

Given above observations and analyses, we empirically recommend using 176 radial views for acquisition acceleration (i.e., equivalent to a sampling factor of 0.5 or an acceleration factor of 4) based on the original protocols with 700 radial views [26]. Compared to actual acquisition times of 144–417 s in original protocols with 700 radial views, the sampling factor of 0.5 leads to an estimated/calculated acquisition time of 47–137 s, i.e., 67% time saving (Table 1).

### Reconstruction time

Although image reconstruction times were not thoroughly tested and recorded, offline reconstruction times were approximately 10 min for data with 352 radial views and 9 min for data with 176 radial views using our proposed method on a desktop computer with an 8-Core i7-6700 CPU at 3.4 GHz (Intel, Santa Clara, CA, USA), 64 GB RAM, and a Quadro P2200 GPU with 5 GB memory (NVIDIA, Santa Clara, CA, USA).

## Discussion

In this study, a multi-echo stack-of-radial MRI method using motion-resolved CS reconstruction with multi-dimensional regularization for free-breathing liver PDFF and ${\mathrm{R}}_{2}^{*}$ quantification was evaluated using retrospectively undersampled data of pediatric patients, with reference results from a previously validated self-gating method with oversampled data of the same subjects.

If not corrected for, respiratory motion during free-breathing acquisitions lead to overestimation of ${\mathrm{R}}_{2}^{*}$ quantification, as well as blurriness or signal attenuation in echo images [23, 24]. Techniques such as self-gating to select data only near end-expiration for reconstruction (the “stable40” method) are effective in compensating for motion artifacts and validated in previous studies [23–26]. However, using only a subset of radial views for reconstruction in the stable40 method causes possible residual undersampling artifacts and inaccurate PDFF or ${\mathrm{R}}_{2}^{*}$ values, also confirmed in the results of this study. Acquiring additional radial views (e.g. oversampling) [23–26] can mitigate this limitation but is not preferred due to prolonged acquisition time (e.g. 1.75–2 times longer).

In this study, our proposed method using motion-resolved CS reconstruction with multi-dimensional regularization addressed these negative effects in an accelerated imaging protocol compared to the stable40 method in previous studies [23–26]. Using reference results from oversampled k-space data, our proposed method demonstrated reduced artifacts and improved PDFF and ${\mathrm{R}}_{2}^{*}$ quantification on undersampled k-space data compared to the stable40 method on the same undersampled k-space data. Some minor residual streaking artifacts originating from the arms may still be present and could potentially be further mitigated or removed using beamforming techniques [38].

Data processing of retrospective undersampling in this study is equivalent to accelerated acquisitions. Using the protocols in this study, an acceleration factor of 4 and an approximate acquisition time saving of 97–280 s (67% shorter) can be achieved, compared to the originally oversampled protocols in pediatric patients [26]. This is a considerable time saving for free-breathing stack-of-radial MRI (typically 2–6 min). Shortening acquisition time may allow for more children to undergo quantitative liver MRI without requiring sedation or anesthesia to restrict bulk motion.

The proposed method was implemented to be flexible to use in both clinical and research settings, supporting both inline reconstruction on the scanner and offline reconstruction. Reconstruction time was approximately 10 min for the recommended protocols in this study. Reconstruction times could be further accelerated by more powerful hardware.

One limitation of this study was that k-space undersampling for acceleration was validated only retrospectively in this study. On the other hand, the golden-angle ordering strategy makes acquisitions of prospective acceleration conveniently achievable by simply reducing the number of radial views without any sequence modifications. It should be noted that the previous study demonstrated the feasibility of prospectively accelerated acquisitions, which were performed and validated in a motion phantom with multiple vials presenting varying ranges of PDFF and ${\mathrm{R}}_{2}^{*}$ [31]. Another limitation was that the number of subjects was relatively small. The PDFF and ${\mathrm{R}}_{2}^{*}$ ranges (2.0%–23.3% and 46.8 s^−1^–497.9 s^−1^, respectively) in this study were relatively limited but still sufficiently large to be representative of pediatric patients [36, 37]. Future prospective studies will evaluate prospectively accelerated acquisitions in a large number of pediatric patients with broader ranges of PDFF and ${\mathrm{R}}_{2}^{*}$ to address these limitations.

## Conclusion

A multi-echo stack-of-radial free-breathing MRI method using motion-resolved CS reconstruction and multi-dimensional regularization for liver PDFF and ${\mathrm{R}}_{2}^{*}$ quantification was evaluated retrospectively in pediatric patients. Using reference results from oversampled k-space data, our proposed method demonstrated reduced artifacts and improved PDFF and ${\mathrm{R}}_{2}^{*}$ quantification on undersampled k-space data compared to a self-gating method. In this study, an acceleration factor of 4 and an approximate acquisition time saving of 97–280 s (67% shorter) can be achieved. This proposed method may allow accelerated free-breathing whole-liver PDFF and ${\mathrm{R}}_{2}^{*}$ mapping in pediatric subjects in approximately 1 min.

## Data Availability

The authors are unable to share the data in this study due to permission restrictions.
